# CUEDC2 down-regulation is associated with tumor growth and poor prognosis in lung adenocarcinoma

**DOI:** 10.18632/oncotarget.3930

**Published:** 2015-05-13

**Authors:** Longhua Sun, Lihong Bai, Gengpeng Lin, Ran Wang, Yangli Liu, Jinghuang Cai, Yubiao Guo, Zhiwen Zhu, Canmao Xie

**Affiliations:** ^1^ Department of Respiratory, The First Affiliated Hospital of Sun Yat-sen University, Institute of Respiratory Diseases of Sun Yat-sen University, Guangzhou, Guangdong, People's Republic of China; ^2^ Department of Pathology, The First Affiliated Hospital of Sun Yat-sen University, Guangzhou, Guangdong, People's Republic of China; ^3^ Respiratory Department, Nanchang Hospital of Integrative Traditional Chinese and Western Medicine, Jiangxi University of Traditional Chinese Medicine Nanchang, Jiangxi, People's Republic of China

**Keywords:** CUEDC2, lung adenocarcinoma, clinical outcome, proliferation, Akt

## Abstract

CUE domain-containing 2 (CUEDC2) is a multi-functional protein, which regulates cell cycle, growth factor signaling and inflammation. We found that CUEDC2 was low in lung adenocarcinoma cell lines and lung adenocarcinoma tissues at both mRNA and protein levels. Low levels of *CUEDC2* were correlated with a shorter survival time in patients with lung adenocarcinoma (*p* = 0.004). CUEDC2 expression was correlated with tumor T classification (*P* = 0.001) at clinical stage (*P* = 0.001) and tumor size (*P* = 0.033). Multivariate analysis suggested that CUEDC2 expression is an independent prognostic indicator for patients with lung adenocarcinoma. Ectopic expression of CUEDC2 decreased cell proliferation *in vitro* and inhibited tumor growth in nude mice *in vivo*. Knockdown of endogenous CUEDC2 by short hairpin RNAs (shRNAs) increased tumor growth. Inhibition of proliferation by CUEDC2 was associated with inactivation of the PI3K/Akt pathway, induction of p21 and down-regulation of cyclin D1. Our results suggest that decreased expression of CUEDC2 contributes to tumor growth in lung adenocarcinoma, leading to a poor clinical outcome.

## INTRODUCTION

Lung cancer is one of the most commonly diagnosed cancer [1–3], Identification and characterization of oncogenes and tumor suppressor genes involved in lung carcinogenesis may reveal new therapeutic targets and prognostic biomarkers.

CUE domain-containing 2 (CUEDC2) is a multi-functional protein that is involved in cell cycle, growth factor signaling and inflammation [4]. The CUE domain, a small ubiquitin-binding motif of about 40 amino acids, can recognize both mono- and poly-ubiquitin and facilitates intermolecular mono-ubiquitination [5, 6]. CUEDC2 interacts with progesterone receptor (PR) and promotes progesterone-induced ubiquitination and degradation of receptors [7].

Also, CUEDC2 is involved in carcinogenesis [7–9]. CUEDC2 is highly expressed in ovarian, brain, kidney and breast tumors [8, 9].

The impact of CUEDC2 expression in lung adenocarcinoma has not been elucidated. In contrast to previous reports that CUEDC2 was highly expressed in many types of cancers, our study suggested that CUEDC2 was markedly down-regulated in lung adenocarcinoma cells and surgical specimens of lung adenocarcinoma.

## RESULTS

### Down-regulation of CUEDC2 in human lung adenocarcinoma cell lines and lung adenocarcinoma tissues

**Figure 1:**
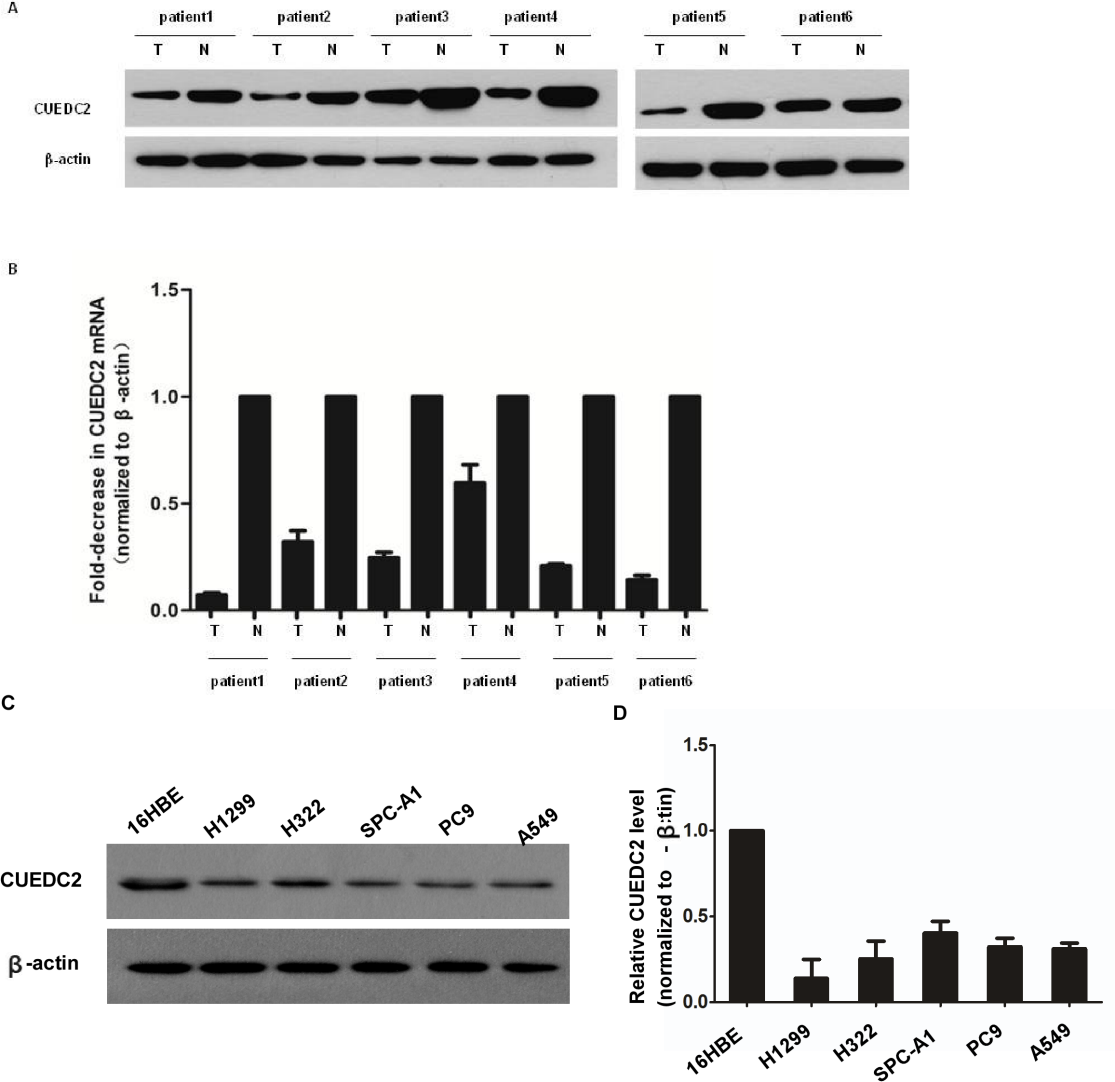
CUEDC2 is down-regulated in lung adenocarcinoma cell lines and lung adenocarcinoma tissues **A.** Western blotting analysis of CUEDC2 protein expression in primary lung adenocarcinoma (T) and paired normal lung (N) tissues from the same patient. **B.** CUEDC2 mRNA levels in lung adenocarcinomas (tumor, T) and matched normal lung tissue (N) from the same patient were analyzed using quantitative real-time PCR (qRT–PCR). The CUEDC2 expression levels were normalized to that of β-actin, the internal control. **C.** Western blotting analysis of CUEDC2 protein expression in A549, PC-9 H322, H1299, and SPC-A1 cells, indicating that CUEDC2 protein levels in lung cancer cells are lower than that in 16HBE cells. **D.** The expression of CUEDC2 mRNA in 16HBE and lung adenocarcinoma cell lines (A549, PC-9 H322, H1299, and SPC-A1) was analyzed using qRT–PCR.

Comparative analysis of CUEDC2 expression was conducted on 6 pairs of matched lung adenocarcinoma tissue and adjacent normal lung tissues. The expression of CUEDC2 protein in three lung adenocarcinoma samples was much lower than in the paired adjacent noncancerous tissue (Fig. 1A). The expression levels of CUEDC2 mRNA (Fig. 1B) were lower in lung adenocarcinoma tumor tissues than in adjacent normal lung tissues from the same patient. Furthermore, Lung cancer cell lines expressed significantly lower levels of CUEDC2 compared to that in the 16HBE cells at both the protein and mRNA levels (Fig. 1C, 1D). Thus, CUEDC2 expression, at both the protein and mRNA levels, was reduced in lung adenocarcinoma cell lines and patient-derived tissues.

### CUEDC2 down-regulation correlates with clinic pathological characteristics and OS of lung adenocarcinoma patients

**Figure 2:**
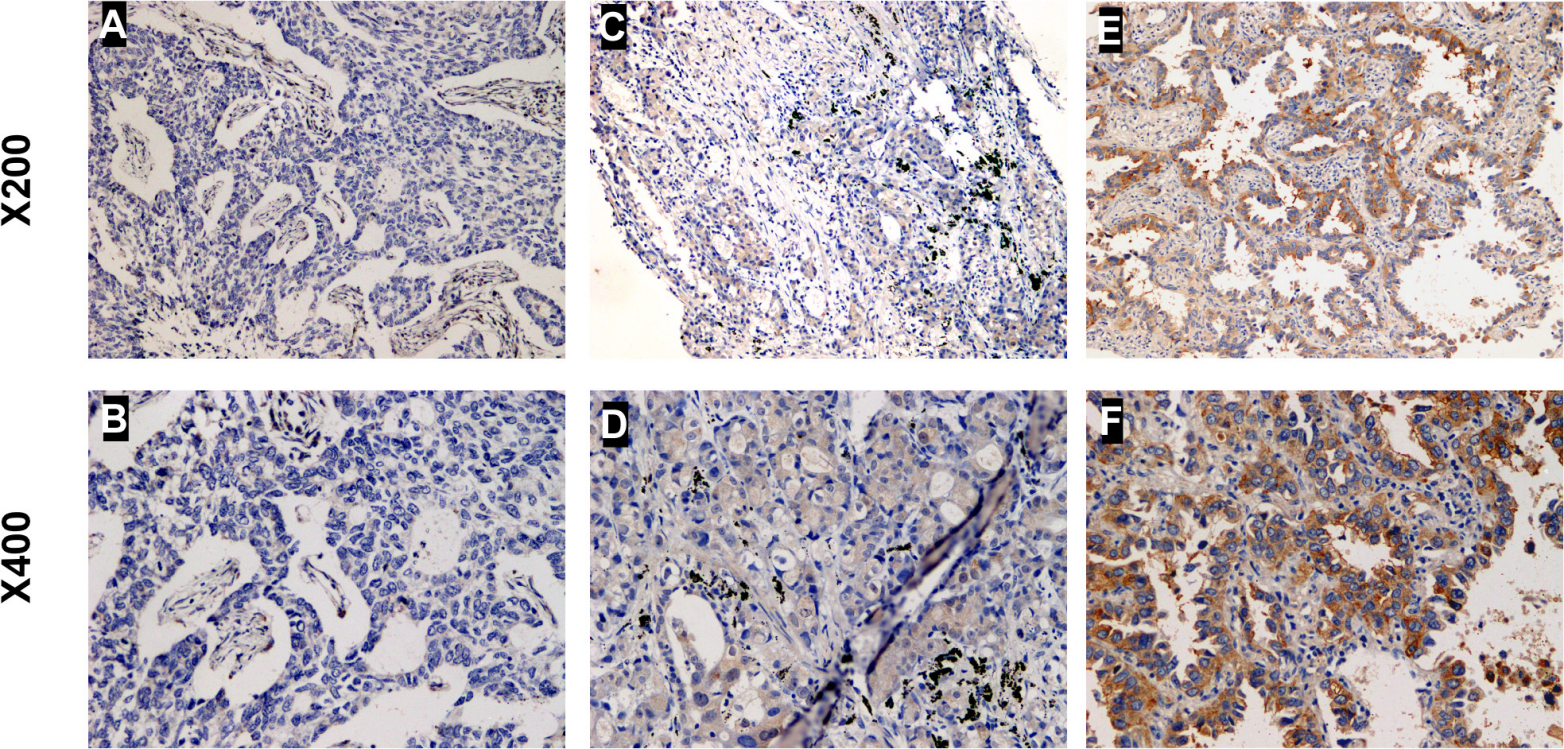
The expression of CUEDC2 protein in lung adenocarcinoma tissues Representative immunohistochemical images of lung adenocarcinoma tissue specimens indicating weakly detectable or undetectable CUEDC2 staining **A and B.** moderate CUEDC2 staining **C and D.** and strong CUEDC2 staining **E and F.** Magnification, × 200 (A, C and E) or × 400 (B, D and F). The CUEDC2 protein was localized mainly to the cytoplasm of cells in the primary lung adenocarcinomas.

**Figure 3:**
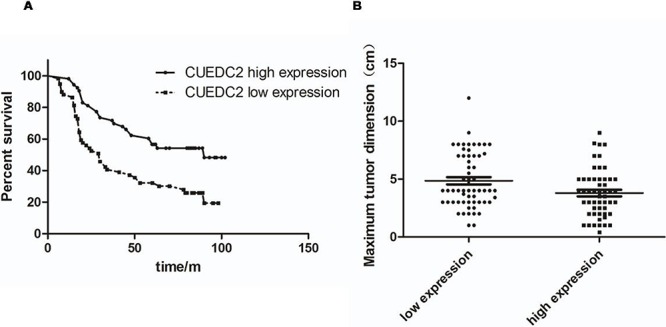
The CUEDC2 protein level affects OS **A.** Kaplan-Meier curves with univariate analysis (Log-rank) for lung adenocarcinoma patients with high CUEDC2 expression (*n* = 53) vs. those with low or undetectable CUEDC2 expression (*n* = 59) for OS (*P* = 0.004). **B.** MTDs of patients with lung adenocarcinoma were significantly different between the high- and low-CUEDC2 expression groups. Each dot represents the MTD of one patient.

Next, we investigated the clinical relevance of CUEDC2 in the proliferation of lung adenocarcinoma. We analyzed CUEDC2 expression in 112 paraffin-embedded, archived lung adenocarcinoma tissues using immunohistochemical staining with an antibody against human CUEDC2. Low expression of CUEDC2 was found in 59 of 112 (52.7%) cases, and high expression of CUEDC2 was detected in 53 of 112 (47.3%) cases (Fig. 2A–2F). Highly-expressed CUEDC2 was mainly localized in the cytoplasm of lung adenocarcinoma cells (Fig. 2A). Furthermore, the expression of CUEDC2 was strongly correlated with the tumor T classification (*P* = 0.001) and clinical stage (*P* = 0.002) of patients with lung adenocarcinoma (Supplementary Table S1). Furthermore, multivariate Cox regression analysis revealed that the CUCED2 expression levels was independent prognostic factors for lung adenocarcinoma patients (Supplementary Table S2). Wilcoxon rank sum test analysis demonstrated that the MTD was significantly different between the high- and low-CUEDC2 expression groups (*P* = 0.033; Fig. 3B). These results supported the hypothesis that CUEDC2 plays an important role in regulating the growth of lung adenocarcinomas. With respect to CUEDC2 expression, the median survival time for patients who had tumors with low expression of CUEDC2 was 31 months, compared with 61 months for patients who had tumors with high expression of CUEDC2 (*P* = 0.004, Log-rank test; Fig. 3A)

Thus, low CUEDC2 expression is a risk factor that predicts poor survival, suggesting that decreased expression of CUEDC2 likely contributes to lung adenocarcinoma cell proliferation and might represent a prognostic biomarker for this disease.

### CUEDC2 is involved in proliferation of lung adenocarcinoma cells

**Figure 4:**
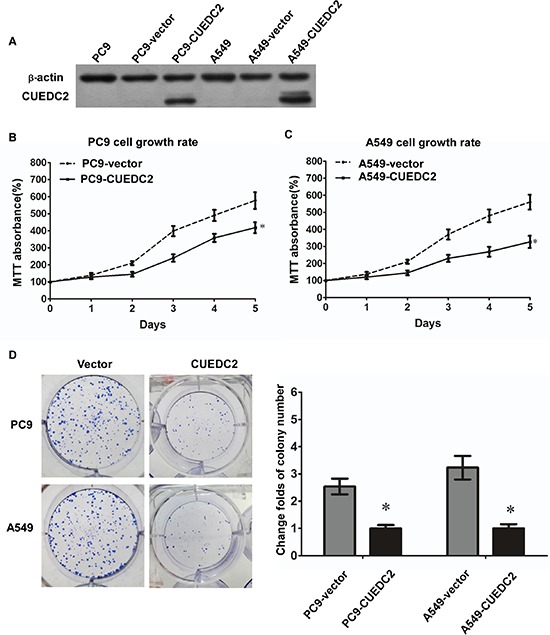
Ectopic over-expression of CUEDC2 decreases proliferation of lung adenocarcinoma cells **A.** CUEDC2 was stably over-expressed in lung adenocarcinoma cell lines and CUEDC2 levels were monitored using western blotting with an anti-CUEDC2 antibody. β-actin was used as the loading control. **B and C.** Assessment of cell proliferation using the MTT assay. **D.** The number of colonies formed by A549-CUEDC2 and PC-9-CUEDC2 cells was markedly less than those formed in the corresponding control cells (A549-Vector and PC-9-Vector cells). Values are expressed as the mean ± SD of three independent experiments, *P < 0.01.

To evaluate the biological role of CUEDC2 in the proliferation of human lung adenocarcinoma cells, we generated lung cancer cells that stably expressed CUEDC2. Stable transfectants derived from two different lung cancer cell lines, A549-CUEDC2 and PC9- CUEDC2, expressed higher levels of CUEDC2 protein compared with the corresponding control cell lines (Fig. 4A) and these two clones were used in subsequent experiments. Compared with the control cells, A549-CUEDC2 and PC-9-CUEDC2 cells showed a significantly decreased rate of cell proliferation, as assessed using the MTT assay (Fig. 4B, 4C), indicating that CUEDC2 could inhibit A549 and PC-9 cell proliferation. Furthermore, colony formation assays indicated that over-expression of CUEDC2 markedly inhibited colony formation of A549 and PC-9 cells (Fig. 4D).

**Figure 5:**
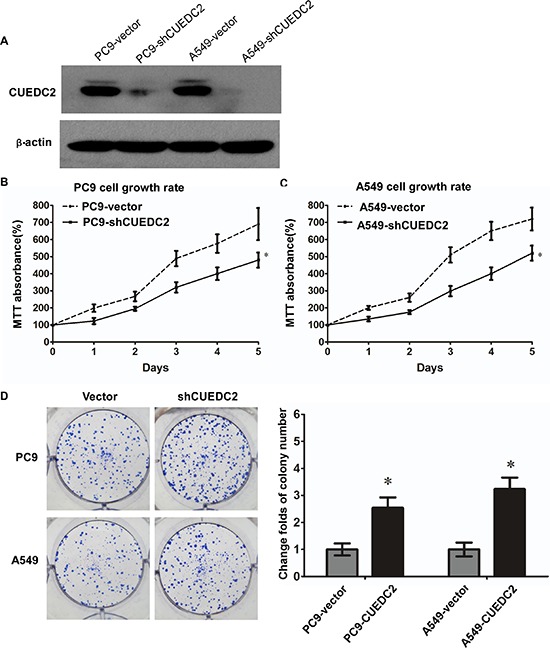
shRNA-mediated depletion of endogenous CUEDC2 increases proliferation of lung adenocarcinoma cells **A.** The CUEDC2 knockdown efficiency in A549/PC9 cells lines was analyzed by western blotting, using an anti-CUEDC2 antibody. β-actin was used as the loading control. **B and C.** MTT assay was performed to monitor cell proliferation. **D.** The number of colonies formed by A549-shCUEDC2 and PC9-shCUEDC2 cells was significantly greater than those formed by the corresponding control cells (A549-Vector and PC-9-Vector cells). Values shown are expressed as the mean ± SD of three independent experiments, *P < 0.01.

In addition, silencing of CUEDC2 expression using an shRNA-mediated method increased the growth of lung adenocarcinoma cells (Fig. 5A), as assessed using the MTT and clone formation assays (Fig. 5B–5D). The colony formation assay showed that knockdown of CUEDC2 markedly increased colony formation of A549 and PC-9 cells. Thus, CUEDC2 suppressed proliferation of lung adenocarcinoma cells.

### CUEDC2 expression arrests lung adenocarcinoma cells at the G1-S phase transition: involvement of cyclin D1, p21 and Akt

**Figure 6:**
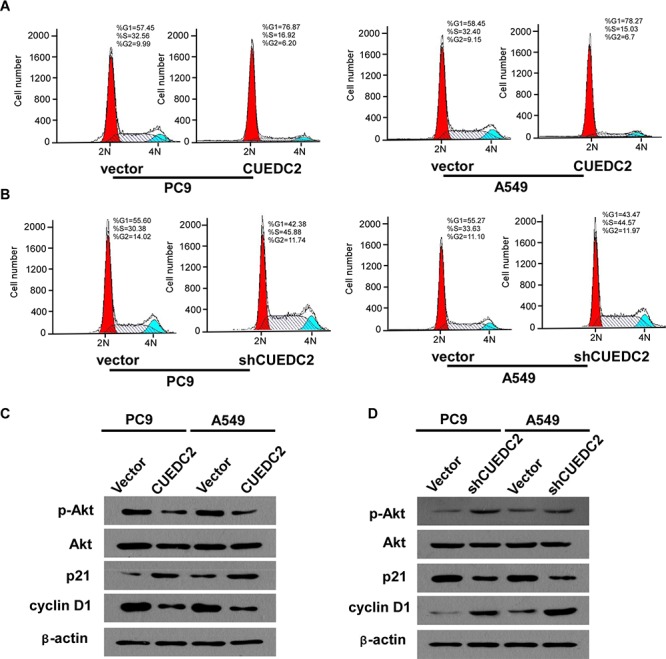
Effects of CUEDC2 on cell cycle arrest and expression of cell cycle regulators in A549/PC9 cells **A and B.** Representative data from the flow cytometry analysis of cell cycle distribution in A549-CUEDC2, A549-shCUEDC2, A549-vector, PC9-CUEDC2, PC9-shCUEDC2, and PC9-vector cells. The percentage of cells in the G0/G1, S, and G2/M phases was quantified and plotted. **C and D.** Western blotting analysis of p-Akt, Akt, p21, and cyclin D1. β-actin was used as the loading control. Values are expressed as the mean ± SD of three independent experiments.

To explore the possible roles of CUEDC2 in inhibiting lung adenocarcinoma cell proliferation, we measured the cell cycle distribution of CUEDC2-over-expressing cells using flow cytometry. The data showed that over-expression of CUEDC2 decreased the percentage of cells in the G0-G1 peak and increased the percentage of cells in the S peak, compared with those in the corresponding control cells (Fig. 6A). shRNA-mediated silencing of CUEDC2 expression significantly decreased the proportion of cells in G1-phase, but elevated the proportion of cells in the S-phase (Fig. 6B). Taken together, these results indicated that the effect of CUEDC2 on anti-proliferation might be due to the arrest of cells at the G1-S transition.

To identify the cell signaling pathways involved in the CUEDC2-mediated cell cycle arrest of A549/PC9 cells, the expression of cell cycle-related proteins was detected using western blotting. The data demonstrated that A549-CUEDC2 and PC9-CUEDC2 cells expressed higher amounts of p21 and lower amounts of cyclin D1 protein, compared with that in the corresponding controls (Fig. 6C). Conversely, down-regulation of CUEDC2 decreased p21 and increased cyclin D1 levels (Fig. 6D). Furthermore, we found that up-regulation of CUEDC2 induces a decrease in the level of phosphorylated Akt (p-Akt) and that down-regulation of CUEDC2 induced an increase in p-Akt levels (Fig. 6C, 6D). Taken together, our results suggested that the phosphoinositol 3-kinase (PI3K)-Akt-p21-cyclin D1 signaling pathway might contribute to the CUEDC2-induced G0/G1-to-S phase cell cycle arrest in lung cancer cells.

### CUEDC2 inhibits tumor growth in nude mice

**Figure 7:**
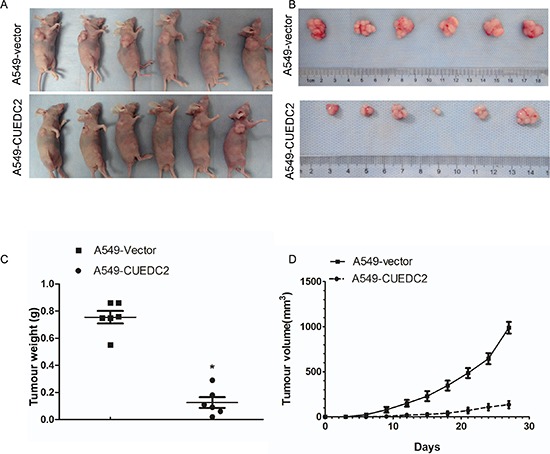
Over-expression of CUEDC2 inhibits tumourigenicity of lung adenocarcinoma cells in nude mice Subcutaneous xenografts of A549-CUEDC2 cells reduced tumor formation 27 d after inoculation **A.** and upon necropsy **B.** compared with the tumors in mice implanted with A549-vector cells. **C.** The weight of the tumors in A549-CUEDC2-injected mice was significantly lower compared with those injected with A549-vector cells. Data represent the mean ± SD of tumor weights from six mice in each group. **D.** The volume of the tumors was significantly lower in mice xenografted with A549-CUEDC2 cells, compared to those xenografted with A549-vector cells. *P < 0.001.

To address the role of CUEDC2 in regulation of tumorigenicity of lung adenocarcinoma cells *in vivo*, A549-CUEDC2 and A549-vector cells were subcutaneously xenografted into the flanks of athymic nude mice and the tumor growth was monitored (*n* = 6 per group). Tumors derived from A549 cells over-expressing CUEDC2 exhibited less growth than the corresponding control cell-derived tumors (Fig. 7A, 7B, 7D). On day 27, the mean weight of tumors induced by A549-CUEDC2 cells was substantially lower than those induced by A549-vector cells (*P* = 0.002; Fig. 7C). Collectively, our results indicate that overexpression of CUEDC2 inhibits lung adenocarcinoma both *in vitro* and *in vivo*.

## DISCUSSION

Here we showed that both mRNA and protein levels of CUEDC2 were significantly down-regulated in human lung adenocarcinoma cell lines and surgically-excised lung adenocarcinoma tumors. CUEDC2 expression was inversely correlated with clinical stage and tumor size in lung adenocarcinoma patients. Ectopic expression CUEDC2 or shRNA-mediated knockdown of CUEDC2 decreased or increased, respectively, cell proliferation, indicating that CUEDC2 inhibits proliferation of lung adenocarcinoma cells. Furthermore, the expression level of CUEDC2 positively correlated with OS of lung cancer patients. Multivariate analysis suggested that CUEDC2 expression is an independent prognostic indicator for survival of lung adenocarcinoma patients. Taken together, our results suggested that low expression of CUEDC2 is associated with tumor growth and that CUEDC2 is an independent prognostic factor for lung adenocarcinoma patients.

We found significant correlation between the tumor T stage and CUEDC2 levels in lung adenocarcinoma tissues from patients. The T stage of the tumor in lung cancer mainly reflects the tumor size, which is an independent prognostic factor for lung cancer patients [10]. Therefore, we analyzed the MTD of the tumors as an additional factor reflective of the T stage. Our results indicated that patients with low expression of CUEDC2 frequently developed larger lung adenocarcinoma tumors, consistent with an anti-proliferative role for CUEDC2 in lung cancer.

A recent study suggested that CUEDC2 inactivates the spindle checkpoint, resulting in chromosome missegregation and aneuploidy [8], which caused neoplasia and preceded the inactivation of tumor suppressor genes at the early stages of tumor formation. Pan X et al [9] showed that CUEDC2 is a determinant of resistance to endocrine therapies in breast cancer and that the over-expression of CUEDC2 in breast cancer increased the possibility of cancer recurrence. Zhang WN et al [11] found that CUEDC2 expression is significantly elevated in human skin cancers and that CUEDC2 degradation is critical for UV light-induced G1 arrest. However, these studies seemed contradictory to others, which showed that CUEDC2 plays an inhibitory role in nuclear factor (NF)-κB and Janus kinase (JAK)1/signal transducer and activator of transcription (STAT)3 signaling [12, 13], while also exerting a cancer-promoting function during tumorigenesis. The NF-κB signaling pathway is involved in inflammation and tumor growth [14]. The JAK1/STAT3 signaling pathway is an oncogenic pathway that is frequently over-activated in human tumors [15, 16]. Therefore, the inhibitory effect of CUEDC2 on NF-κB and JAK1/STAT3 is likely to prevent tumorigenesis, rather than promoting tumor growth. Furthermore, in our study, we provided evidence that in lung adenocarcinoma, CUEDC2 expression is down-regulated, and that CUEDC2 inhibits growth of lung adenocarcinoma cells both *in vitro* and *in vivo*. We concluded that CUEDC2 plays a tumor-suppressive role in lung adenocarcinoma. These findings indicated that CUEDC2 has a dual function, either in tumor promotion or tumor suppression. However, it needs to be emphasized that the function of CUEDC2 appears to be different in different types of cancers. Further studies are needed to elucidate the molecular mechanisms through which CUEDC2 exerts these disparate effects in different cancers.

Zhang PJ et al [7] found that CUEDC2 could inhibits breast cancer cell growth by decreasing mitogen-activated protein kinase activity. Our results demonstrate that over-expression of CUEDC2 inhibits Akt in lung adenocarcinoma cells. The PI3K/Akt pathway is involved in apoptosis, survival, proliferation, differentiation and cancer including endometrial and non-small cell lung cancer [17–21]. PI3K/AKT signaling regulats p21 expression, which is involved in cell cycle regulation [22–24]. Cyclin D1 is a well-established human oncogene [25], which is over-expressed in lung cancer, breast cancer, melanoma and pancreatic cancer [25–28]. Knockdown of cyclin D1 protects mammary tissues from malignant transformation [29]. Gautschi O et al [30] have indicated that cyclin D1 overexpression is involved in malignant transformation in lung tissues.

Our results demonstrated that up-regulation of CUEDC2 decreases proliferation of lung adenocarcinoma cells, by inhibiting the PI3K-Akt transduction pathway and concomitant p21 up-regulation and cyclin D1 down-regulation. Conversely, down-regulation of endogenous CUEDC2 significantly increased growth of lung adenocarcinoma cells by activating the PI3K-Akt singling pathway, down-regulating p21, and up-regulating cyclin D1. These data suggest that the CUEDC2 affects lung cancer cell growth through the PI3K-Akt-P21-cyclin D1 singling pathway.

In summary, our data show that CUEDC2 functions as a putative tumor suppressor in lung adenocarcinoma, and that the anti-proliferative effects of CUEDC2 are mediated through its effects on the PI3K/Akt signaling pathway.

## MATERIALS AND METHODS

### Patients and tissue specimens

Lung adenocarcinoma and matched adjacent normal lung tissue samples used in this study were obtained during surgical removal of tumors from patients histopathologically diagnosed with lung adenocarcinoma at the Department of Pathology, The First Affiliated Hospital of Sun Yat-sen University from January 1, 2002 to January 1, 2007. The histological characterization and clinicopathological staging of the samples were performed according to World Health Organization criteria [31] and the current International Association for the Study of Lung Cancer tumor-node-metastasis (TNM) classification or International Union Against Cancer TNM classification [32]. Detailed clinic pathological data for all patients, including their age, clinical stage, T classification, N classification and distant metastasis status, is presented in Supplementary Table S3. Written informed consent was provided by all patients for use of their tissue specimens and medical data for research purposes. The study was approved by the Institutional Review Board of Sun Yat-sen University.

### Plasmid constructs

To construct the pBabe-retro-puro-Flag-CUEDC2 expression vector, the full-length CUEDC2 cDNA fragment was generated by PCR from a human mammary cDNA library and inserted into the pBabe-retro-puro vector. The pSUPER-retro short hairpin RNA (shRNA) retroviral vector expressing CUEDC2 siRNA (target sequence: 5′-GAAGCTGATCCGATACATC-3′; 5′- GTACATGATGGTGGATAGC-3′) were constructed by recombinant DNA technology as described previously [12]. The pBabe-retro-puro and pSUPER-retro plasmids were generated in collaboration with the laboratory of Dr. Meng-Feng Li at Sun Yat-Sen University.

### Constructions of stable cell lines

The Phoenix packaging cells were transfected with the pSUPER-retro or pSUPER-retro CUEDC2 shRNA retroviral vector using a liposome-based transfection method and the packaged virus-containing supernatant was collected and used to infect A549 and PC9 cells. Stable integrants were selected using Puromycin for 2 weeks. Additionally, A549 and PC9 cell lines stably expressing CUEDC2 were infected with the virus-containing supernatant from Phoenix cells transfected with pBabe-retro-puro or pBabe-retro-puro-Flag-CUEDC2. Stable integrants were selected with 0.2 μg/mL puromycin for 2 weeks as described previously [13]. The cells stably expressing CUEDC2 or carrying the vector alone were designated as “-CUEDC2” or “-Vector” cells, while those stably expressing the CUEDC2-specific shRNA were designated as “-shCUEDC2” cells.

### Colony formation assay

Colony formation assays were performed as described previously [33]. Briefly, a total of 400 cells were plated onto 60 mm plates and incubated at 37°C in a 5% CO_2_ incubator for 14 days. Fresh medium was added every 4 days. At the end-point, the cells were washed with cold PBS, fixed with 4% paraformaldehyde for 30 min and stained with 1% crystal violet solution for 20 min at room temperature. Three independent trials of the experiment were carried out for each cell line.

### Flow cytometry analysis

Flow cytometry was performed according to a standard method, as described previously [34]. Cells were harvested by trypsinization, washed in ice-cold phosphate-buffered saline (PBS) and fixed in 80% ice-cold ethanol in PBS. Before staining, the cells were centrifuged in a cooled centrifuge and resuspended in cold. Bovine pancreatic RNase (Sigma-Aldrich) was added at a final concentration of 2 μg/mL, and the cells were incubated at 37°C for 30 min, followed by incubation in 20 μg/mL propidium iodide (Sigma-Aldrich) for 20 min at room temperature. The cell cycle distribution was analyzed using FACScan cytometry (Becton-Dickinson, San Jose, CA, USA). Three independent trials of the experiment were carried out for each cell line.

### *In vivo* tumor xenograft model

BALB/c-nu mice (5–6 weeks of age, 18–20 g) were purchased from the Centre of Experimental Animal of Sun Yat-Sen University and were housed in barrier facilities on a 12 h light/dark cycle. All experimental procedures were approved by the Institutional Animal Care and Use Committee of Sun Yat-Sen University. The BALB/c nude mice were randomly divided into two groups (*n* = 6 per group). For tumor cell implantation, A549-CUEDC2 and A549-vector cells (5 × 10^6^) were trypsinized and resuspended in 0.25 mL PBS and subcutaneously injected into nude mice. After development of palpable tumors, the tumor volume (V) was monitored every 2 days and calculated using the formula: V = 0.5 × length × width^2^. Twenty-seven days after inoculation, the mice were anesthetized and sacrificed, and the tumors were removed and weighed.

### Statistical analysis

All experimental data were analyzed using the SPSS 17.0 statistical software package. For survival analysis, a cohort of 112 patients with lung adenocarcinoma was divided into two groups based on the CUEDC2 expression level; the low-CUEDC2 expression group (with a staining index score ≤ 4) and the high-CUEDC2 expression group (with a staining index score > 4). Kaplan–Meier analysis and the Log-rank test were used to assess the differences between the two groups. Survival data were evaluated using univariate and multivariate Cox regression analysis. The Chi-square test was performed to analyze the correlation between clinicopathologic characteristics and CUEDC2 expression. Differences in the maximum tumor diameter (MTD) between the two groups were analyzed using the Wilcoxon rank sum test. The *in vitro* and *in vivo* observations were analyzed using the Student's *t*-test or one-way analysis of variance. The data from at least two independent experiments were expressed as the mean ± standard deviation (SD). In all cases, values of *P* < 0.05 were considered statistically significant.

## SUPPLEMENTARY DATA


