# Supplementary material for: CUEDC2 down-regulation is associated with tumor growth and poor prognosis in lung adenocarcinoma
Source: Oncotarget. 2015 May 13;6(24):20685–96. doi: 10.18632/oncotarget.3930 (PMC4653035; doi:10.18632/oncotarget.3930)
Supplement: Supplementary file 1 [file oncotarget-06-20685-s001.pdf]

SUPPLEMENTARY DATA

Cell lines and cell culture

A549, PC9, SPC-A1, H1299, and H322 human lung adenocarcinoma cell lines and an immortal human bronchial epithelial cell line (16HBE) were used in this study. A549 and PC9 cells were obtained from Cell Bank, Chinese Academy of Sciences (Shanghai, China) and maintained using routine methods in our laboratory. SPC-A1, H1299, H322, and HBE cells were generously provided by Prof. Liantang Wang at Department of Pathology, The First Affiliated Hospital of Sun Yat-Sen University. Cells were maintained in RPMI 1640 medium (Gibco-BRL, Carlsbad, CA, USA) supplemented with 10% fetal bovine serum (Gibco) at 37°C in a humidified incubator in an atmosphere of 5% CO_2.

Quantitative real-time PCR (qRT-PCR)

Total RNA was isolated from the cells using Trizol reagent (Invitrogen, Carlsbad, CA, USA) according to the manufacturer's instructions. Quantitative real-time PCR (qRT–PCR) was carried out using the FastStart Universal SYBR Green Master (ROX; Roche, Toronto, ON, Canada) on the ABI StepOne plus PCR detection system. The β-actin gene was used as the reference gene. The sequences of the primer pairs were as follows: CUEDC2, Forward (F), 5′-CTGAGCGATGCCAGGAAC-3′ and Reverse (R), 5′-CCGAACAGGTAGGGAACAC-3′; β-actin, F, 5′-CGCCAGCTCACCATGGATGATGAT-3′ and R, 5′-TCTCTTGCTCTGGGCCTCGTCG-3′. Each sample was analyzed in triplicate and the mean expression level was calculated.

Western blot analysis

Total protein was extracted from cells using a lysis buffer and the protein concentration was measured using the BCA Protein Assay kit (CWBiotech, Beijing, China). Equal amounts of protein were separated by sodium dodecyl sulfate-polyacrylamide gel electrophoresis on 10% polyacrylamide gels. After electrophoretic transfer to a polyvinylidene difluoride membrane, nonspecific binding sites were blocked with 5% nonfat milk in Tris-buffered saline containing 0.1% Tween 20 (TBST) followed by incubation with primary antibodies overnight at 4°C. After three washed with TBST, the membranes were incubated with anti-rabbit or anti-mouse horseradish peroxidase-conjugated secondary antibodies (1:5000). Protein bands were detected using enhanced chemiluminescence (Millipore, Billerica, MA, USA).

Western blotting was performed as described previously 1 with anti-human CUEDC2 (Abcam), anti-p21Cip1, anti-phospho-AKT, anti-AKT (all from Cell Signaling Technology), and anti-cyclin D1 (Epitomics) antibodies. The membranes were stripped and re-blotted with an anti-β-actin monoclonal antibody (Sigma, St Louis, MO, USA) as a loading control. The Western blotting assay was performed at least three independent times with similar results.

Immunohistochemistry (IHC)

IHC analysis was performed with a human CUEDC2 antibody (Abcam) to assess protein expression in 112 paraffin-embedded lung adenocarcinoma tissues. The details of the IHC procedures were as described in a previous report 2. In brief, IHC was performed using a standard streptavidin-biotin-peroxidase complex method (EnVision™ Detection Systems; Dako, Copenhagen, Denmark). The tissue sections were incubated with a polyclonal rabbit anti-human CUEDC2 antibody (Abcam) overnight at 4°C. A semi-quantitative scoring criterion for CUEDC2 IHC was used, in which both the staining intensity and the numbers of positive cells were taken into account. A staining index (with values from 0-12), obtained as the intensity of CUEDC2-positive staining (scored as follows: negative = 0, weak = 1, moderate = 2, or strong = 3) and the proportion of cells of interest that were stained positive (scored as follows: <25% = 1, 25-50% = 2, >50% to <75% = 3, ≥75%=4) were calculated.

The degree of immunostaining of formalin-fixed, paraffin-embedded sections was reviewed and scored independently by two pathologists, based on both the proportion of positively-stained tumor cells and the intensity of staining. Cutoff values to define high and low expression of CUEDC2 were chosen according to a measure of heterogeneity using the Log-rank test with respect to overall survival (OS). An optimal cutoff value was identified. A score >4 was used to define tumors with high expression and a score ≤4 as tumors with low expression of CUEDC2.

Cell proliferation assay

A549-CUEDC2, A549-Vector, A549-shCUEDC2, PC9-CUEDC2, PC9-Vector, and PC9-shCUEDC2 cells were plated on 96-well plates (Corning, NY, USA) and allowed to attach overnight. After 24h, 48h, 72h, 96h and 120h, 20 μL of 5 mg/mL MTT (Sigma, St. Louis, MO, USA) solution was added to each well and the plates were

incubated for 4 h at 37°C. After the media was removed, 200 µL of DMSO (Sigma) was added and the absorbance at 490 nm was measured using a micro- plate reader.

Transient transfection

Cells were seeded in a 6-well plate 24h before transfection. The p21 siRNAs (Ribobio, Guangzhou, China) and cyclinD1 expression vector (Genecopoeia) were transfected at a concentration of 50 nmol/L each with Lipofectamine 2000 (Invitrogen) according to the manufacturer's instructions. The target sequences of p21 siRNAs are as follows: forward: 5′-CCUCUGGCAUUAGAAUUAU dTdT- 3′, reverse: 3′-dTdT GGAGACCGUAAUCUUAAUA -5′.

Colony formation assay

After 48 h p21 siRNA and cyclinD1 expression vector transfection, a total of 400 cells were plated onto 60 mm plates and incubated at 37°C in a 5% CO_2 incubator for 14 days. Fresh medium was added every 4 days. At the end-point, the cells were washed with cold PBS, fixed with 4% paraformaldehyde for 30 min and stained with 1% crystal violet solution for 20 min at room temperature. The visible colony numbers were counted; the experiment was performed in triplicate.

Cell cycle analysis

Cells were seeded in six-well plates and incubated overnight until 30–50% confluent, then transfected with 50 nM p21 siRNA and cyclinD1 expression vector. The cells were harvested at 48 h after transfection, washed in cold PBS, fixed with 80% ethanol for 24 h at 4°C, then stained with propidium iodide buffer (50 mg/ml propidium iodide, 0.1% sodium citrate and 0.1%Triton X-100) for 30 min at room temperature. The cellular DNA was analysed using a FACScan (Becton-Dickinson Immunocytometry Systems, San Jose, CA, USA). Cell Quest software was used. This experiment was performed in triplicate.

REFERENCES

1. Li W, Yu CP, Xia JT, et al. Sphingosine kinase 1 is associated with gastric cancer progression and poor survival of patients. Clin Cancer Res. 2009; 15:1393–9.
2. Li J, Zhang N, Song LB, et al. Astrocyte elevated gene-1 is a novel prognostic marker for breast cancer progression and overall patient survival. Clin Cancer Res. 2008; 14:3319–26.

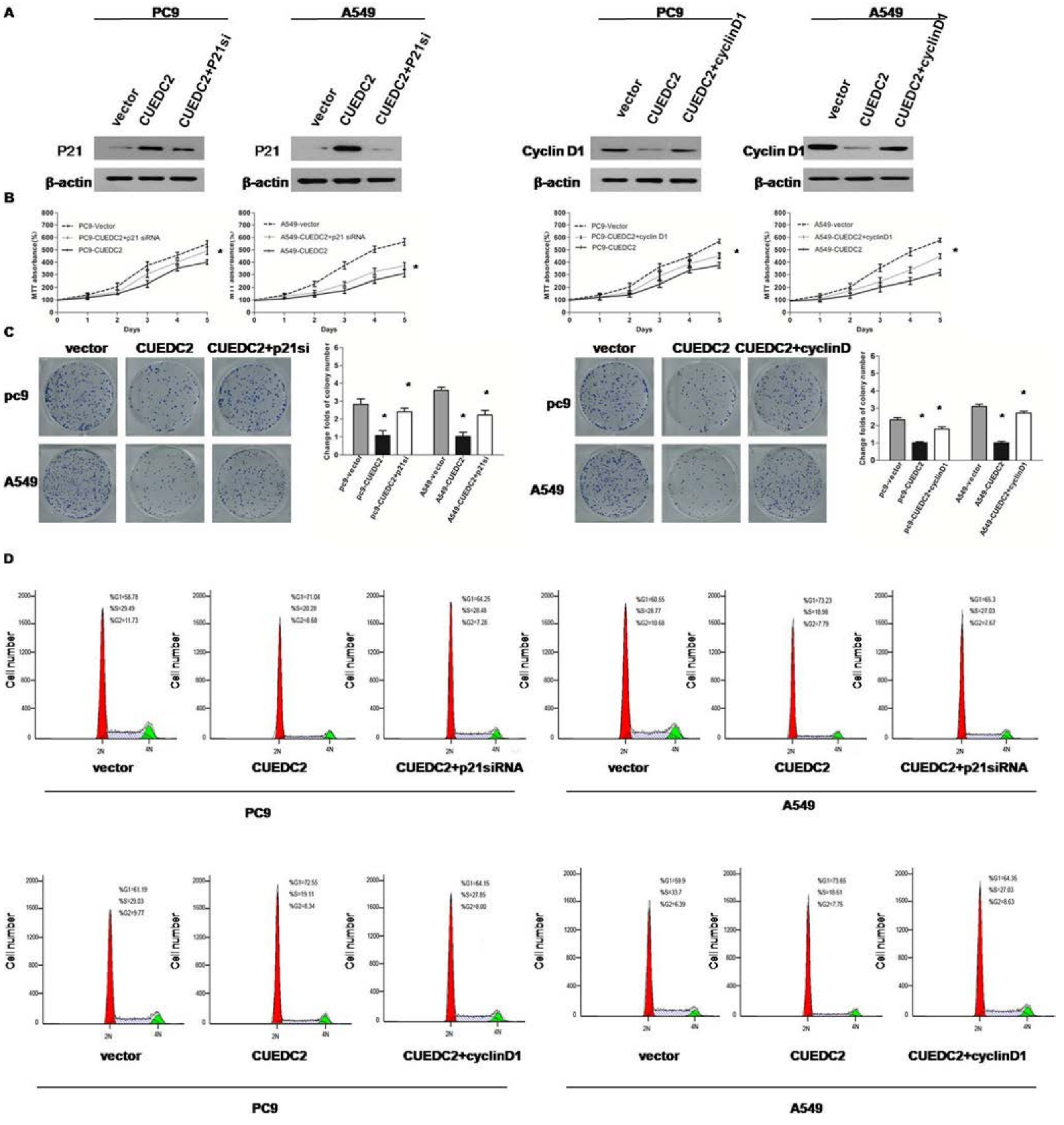

Supplementary Figure S1: Overexpression of CUEDC2 suppressed lung adenocarcinoma cell proliferation through p21 and cyclinD1. A. Western blotting of p21 , cyclinD1 expression in indicated cells. β-actin was used as a loading control. **B.** MTT assays revealed cell growth curves of indicated cells. **C.** representative micrographs (left) and relative quantification (right) of crystal violet-stained cell colonies analyzed by colony formation Assay. **D.** flow-cytometric analysis shows the proportion of indicated cells after 48 hours transfection with p21siRNA or cyclinD1 expression vector.

Supplementary Table S1. Correlation between CUEDC2 expression and clinicopathologic characteristics of lung adenocarcinoma patients

Characteristics	CUEDC2 expression			
	n	Low or none	High	Chi-square test
		No. cases (%)	No. cases (%)	*p* value
**Gender**				0.695
Male	71	36	35	
Female	41	23	18	
**Age**				0.335
≤65	69	39	30	
>65	43	20	23	
**Clinical Stage**				**0.002***
I	51	17	34	
II	30	19	11	
III	25	19	6	
IV	6	4	2	
**T classification**				**0.001***
T1	40	13	27	
T2	28	26	22	
T3	13	11	2	
T4	11	9	2	
**N classification**				0.123
N0	113	35	36	
N1	17	8	9	
N2	20	15	5	
N3	4	1	3	
**M classification**				0.682
M0	106	55	51	
M1	6	4	2	
**Pathologic Differentiation**				
Well	42	25	17	0.530
Moderately	64	31	33	
Poorly	6	3	3	
**Vital status (at follow-up)**				
Alive	68	44	24	**0.002***
Death	44	15	29	

*$p < 0.05$

Supplementary Table S2. Univariate and multivariate analysis for overall survival (Cox proportional hazards regression model)

Variable	Univariate		Mutivariate analysis		
	No.	*p* value	HR	95% CI	*p*value
**CUEDC2**		<0.001	0.402	0.237-0.682	**0.001***
low expression	59				
high expression	53				
**Age**		0.512	0.858	0.617-1.423	0.761
≤65	69				
>65	43				
**Gender**		0.818	0.868	0.576-1.308	0.499
Male	71				
Female	41				
**T classification**		<0.001			
T1+T2	88				
T3+T4	24				
**N classification**		0.001			
N0	71				
N1-3	41				
**M classification**		<0.001			
M0	106				
M1	6				
**Differentiation**		0.001			
Well	42				
Moderately/poorly	70				
**Clinical Stage**		<0.001	1.632	1.282-2.078	<**0.001***
I-II	81				
III-IV	31				

HR: Hazard Ratio; CI, confidence interval $^{*}p < 0.05$

Supplementary Table S3. Clinical data of samples of lung adenocarcinoma

Characteristics	Number of cases (%)
**Gender**	
Male	71
Female	41
**Age**	
≤65	69
>65	43
**Clinical Stage**	
I	51
II	30
III	25
IV	6
**T classification**	
T1	40
T2	48
T3	13
T4	11
**N classification**	
N0	71
N1	17
N2	20
N3	4
**M classification**	
M0	106
M1	6
**Pathologic Differentiation**	
Well	6
Moderately	64
Poorly	42
**Expression of CUEDC2**	
Low expression	59
High expression	53
